# Genetic Analysis of a Collection of Rice Germplasm (*Oryza sativa* L.) through High-Density SNP Array Provides Useful Information for Further Breeding Practices

**DOI:** 10.3390/genes13050830

**Published:** 2022-05-06

**Authors:** Ping Huang, Qiongyao Gu, Yong Hu, Huahui Li, Zhigang Wu, Weihua Liu, Zhenhua Zhu, Pingrong Yuan, Liu Duan, Ying Zhou, Hanyu Luo, Shuyan Kou, Li Liu

**Affiliations:** 1 Institute of Food Crops, Yunnan Academy of Agriculture Sciences, Kunming 650205, China; tmhping@163.com (P.H.); qiongyaogu413@163.com (Q.G.); lihuahuiweida@163.com (H.L.); wuzhigangswu@163.com (Z.W.); lwhynu@126.com (W.L.); zhzhh86@126.com (Z.Z.); yuanpr2003@aliyun.com (P.Y.); 2 State Key Laboratory of Biocatalysis and Enzyme Engineering, Hubei Collaborative Innovation Center for Green Transformation of Bio-Resources, Hubei Key Laboratory of Industrial Biotechnology, School of Life Sciences, Hubei University, Wuhan 430062, China; yonghu@hubu.edu.cn (Y.H.); duanliu28@163.com (L.D.); 3 College of Life Science and Health, Wuhan University of Science and Technology, Wuhan 430065, China; zhouying0613@wust.edu.cn; 4 Yunnan Inntra Intelligence System Co., Ltd., 22/F, Junhao building, Kegao Road High-Tech Zone, Kunming 650101, China; luohyinntra@163.com

**Keywords:** genetic diversity, rice, SNP, local variety

## Abstract

Traditional breeding strategies mainly focus on the evaluation of trait performance, but pay less attention to the changing genetic background. A comprehensive understanding of the genetic diversity of germplasms is crucial for the deliberate improvement of specific traits. A collection of 154 highland rice varieties were collected as the initial genetic resource in our breeding program to improve the pathogen resistance and eating and cooking qualities. These varieties were analyzed using a whole-genome SNP array and were clustered into three groups. Further analysis revealed that the favorable alleles of pathogen resistance genes are mostly absent in our collected varieties. However, it showed that most varieties possess favorable alleles of *Waxy* (*Wx*) and *ALKALI DEGENERATION* (*ALK*), which are able to enhance the eating and cooking qualities. Moreover, only about one fifth of all varieties harbors favorable the allele of fragrance gene *Betaine* *aldehyde dehydrogenase* (*BADH2*). Together, these results give an overall view of the genetic constitution of the target traits, which provide useful information for future genetic improvement in breeding practices.

## 1. Introduction

Rice (*Oryza sativa* L.) is one of the most important staple crops that feeds approximately half of the population in the world [1]. It has been domesticated to be adapted to different cultivation regions with diverse environmental conditions [2,3,4]. The Yunnan province, located in southwestern China, is one of the major rice-producing areas, where rice terraces are the local specific cultivation mode. The cultivation region expands in the vertical direction, which ranges from areas with hundreds of meters up to >2500 m [5]. Moreover, there are 26 ethnic minorities in the Yunnan province. Different groups maintain and grow their own rice landraces for highly specific uses (e.g., ethnic dietary customs, medicinal uses, festivals, and religious ceremonies) [6]. Therefore, the local rice varieties adapted to the diverse environment conditions or used for specific demands provide an excellent opportunity for studies of the genetic diversity of local rice varieties.

Because of the high density and diverse detection methods, single nucleotide polymorphism (SNP) markers have gradually become the ideal markers used for genetic discovery research and molecular breeding. The investigation of the rice genome has revealed millions of SNPs [7,8,9,10]. Recent progress in next-generation sequencing technology boosted the establishment of an SNP marker-based platform for genotyping [11,12]. SNP array technology provides a low-cost and efficient method to analyze the genotypes of multiple loci in the whole-genome scale [13,14,15,16]. Furthermore, SNPs from cloned genes provide useful information for breeding applications [17,18,19].

In recent years, consumer preferences have shifted towards rice with better eating and cooking qualities. Although each country or region have a particular preference of rice with a specific combination of quality traits, a common trait across most countries in Asia is the soft texture of freshly cooked rice, and its maintenance during storage [20]. This trait is highly related to the amount of amylose in the grain, which is controlled by two major loci, *Waxy* (*Wx*) and *ALKALI DEGENERATION* (*ALK*)/*starch synthase IIa* (*SsIIa*) [21,22,23]. Previous studies have revealed a relationship between the genetic variations within loci and the change in phenotypes. For example, *Wx^b^* in Nipponbare is a weak allele conferring a moderate amount of amylose accumulation and better quality [24], and *ALK* in Minghui 63 is an unfavorable allele that increases the gelatinization temperature and results in a lower eating quality [21]. Furthermore, fragrant rice is gaining widespread popularity among consumers worldwide. The recessive allele of *Betaine* *aldehyde dehydrogenase* (*BADH2*), *badh2.1* is associated with the rice fragrance [25]. SNP mutation within *badh2.1* was used as functional nucleotide polymorphism for both breeding and further research.

In this research, a total number of 154 cultivated rice varieties were genotyped with 44,263 high-quality SNP markers throughout all 12 chromosomes. The genetic structure of these varieties was analyzed. By using the SNP markers located in the cloned and characterized genes [26], especially the genes with regard to pathogen resistance and amylose content, the genic characteristics of bio-stress resistance and rice eating quality were evaluated. Our results not only reveal the complex genetic diversity in rice varieties in Yunnan, but also provide useful information for future breeding practices.

## 2. Materials and Methods

### 2.1. Plant Materials and Growing Conditions

A total number of 154 local rice germplasms from the Yunnan province, China, together with 3 wild rice accessions (*Oryza rufipogon*, *Oryza meyeriana* and *Oryza officinalis*) and Nipponbare as the control variety were analyzed in this study. All the materials were planted in the experimental plot located in the Songming county, Kunming city, Yunnan province, China. The longitude and latitude were 102.41 E and 25.05 N, respectively. The sowing date was March 17th 2019, and the transplanting date was between the 14th and 16th of May.

### 2.2. DNA Isolation

Flag leaves of each variety were collected for DNA isolation. DNA was extracted from the frozen leaf tissues of each variety through the CTAB (Cetyltrimethylammonium bromide) method [27].

### 2.3. SNP Array Analysis and Phylogenetic Tree Construction 

A total of 44,263 high-quality SNP markers (GenTrain score > 0.5, missing rate < 20%, heterozygous rate < 0.05, and minor allele frequency (MAF) > 0.05) were obtained after genotyping the germplasm collection of 4726 cultivated rice. The distances between any two lines were measured by the ratios of polymorphic SNPs. The phylogenetic tree was constructed based on an improved version of the neighbor-joining algorithm method [28] using the Rlape software [29]. A Manhattan plot was generated by a costumer R script.

### 2.4. Background Analysis

The genotypes of rice varieties were visualized using a program written in R scripts. The program translated the SNP data information into color bars according to the length of chromosomes. Chromosome fragments with homozygous SNP genotypes are shown as empty bars with a green border, whereas those with heterozygous SNP genotypes are shown as solid blue lines or bars.

### 2.5. Functional Analysis of Target Genes

The SNP array platform was designed to cover the causal SNP(s) for most cloned rice genes according to the information in public publications [26]. To determine the genotype of a specific gene, the genotype of a causal SNP or the haplotype represented by a combination of a group of SNPs were used to compare with a standard genotype represented by the indicated variety. The favorable allele of a gene means either the gene confers disease resistance or enhances the eating and cooking qualities.

## 3. Results

### 3.1. The Characteristics of Whole-Genome SNP Array

**Figure 1:**
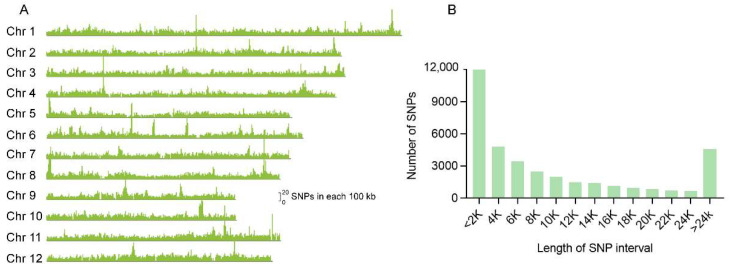
Characteristics of the SNPs used for array establishment in the whole genome of rice. (**A**) Distribution of SNPs along the 12 chromosomes. The height of the peaks indicates the numbers of each 100 kb region. (**B**) The frequency distribution diagram for the distance between the SNPs.

A SNP array platform was designed with a total number of 44,263 high-quality SNP markers to cover the entire rice genome. These SNPs are almost evenly distributed on the 12 chromosomes with an average of 11 SNP markers per 100 kb (Figure 1A). The gaps between two adjacent SNPs were calculated (Figure 1B), most of which are shorter than 25 kb. The gaps of nearly a third of all SNPs are shorter than 2 kb, and about 83.7% of which are shorter than 20 kb.

### 3.2. The Genetic Structure of the Local Varieties in Yunnan

**Figure 2:**
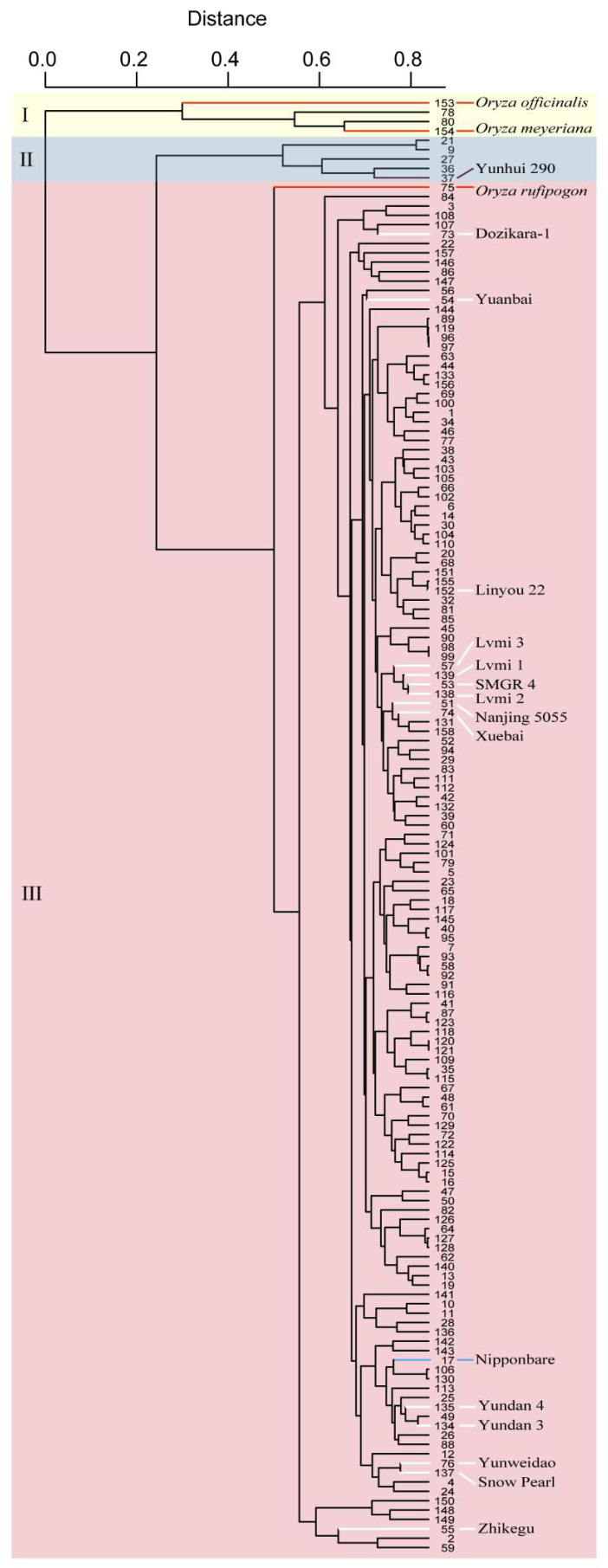
The cluster results of the local rice varieties in Yunnan based on SNP genotypes at the whole-genome level. Groups I, II and III are indicated with a light yellow, light blue and light red background, respectively. Varieties indicated with red and white lines are the wild rice accessions and introduced varieties, respectively. The control variety Nipponbare is indicated with a blue line. Yunhui 290, a classical *indica* inbred variety, is indicated with a purple line.

The collection comprised 154 local varieties together with 3 wild rice accessions (*Oryza rufipogon*, *Oryza officinalis* and *Oryza meyeriana*) and the control variety Nipponbare and was subjected to whole-genome analysis with an SNP array. Based on the genotypes of the SNP markers, these varieties were clustered into three groups, named as groups I, II and III (Figure 2). Wild rice accessions and two other varieties, Fengdao 11 (No. 78) and Fengdao 15 (No. 80), were grouped together and composed group I. Group II, which comprised five local varieties, together with the varieties of group I showed distinct genetic diversities compared with the other varieties in group III. A total of 94.2% of all tested varieties make up group III, within which Nipponbare is listed. 

In the breeding course, several elite varieties from other cultivation regions were introduced in the Yunnan province. Parts of these varieties, such as Dozikara-1, Yuanbai, Linyou 22 and Zhikegu, were grouped together with different local varieties, which implies that gene resource from these varieties were introduced in local varieties during the breeding progress (Figure 2). However, another group of introduced elite varieties comprised Lvmi 3, Lvmi 1, SMGR 4, Lvmi 2, Nanjing 5055 and Xuebai 2, which suggests that they share a similar genetic background, but it is not well used in the breeding progress (Figure 2).

### 3.3. The Background Analysis of the Local Varieties in Yunnan

**Figure 3:**
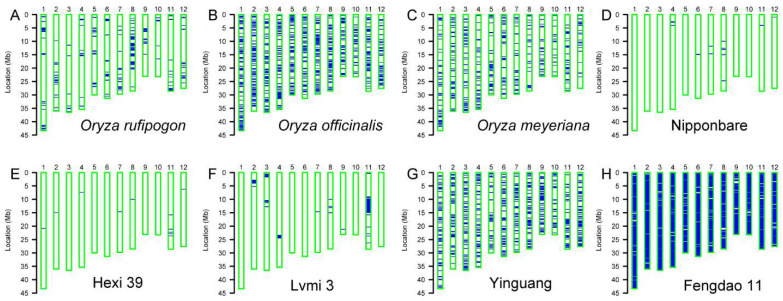
Background analysis with whole-genome SNP array. The background map of wild rice accessions (**A**–**C**), the control variety Nipponbare (**D**) and several representative varieties (**E**–**H**). The 12 chromosomes are indicated with blue bars. Empty bars with a green border represent the homozygous background, and blue lines or bars represent the heterozygous fragments.

The genetic background of parent lines in breeding crosses provides very important information to further breeding practices. Taking advantage of the whole-genome SNP array, the background of 154 local varieties, two wild rice accessions and the control plant Nipponbare was analyzed. Wild rice accessions exhibited heterogeneous backgrounds since they showed an outcrossing character [30]. Similarly, large portion of the chromosome fragments in our wild rice accessions (*Oryza officinalis*, *Oryza meyeriana* and *Oryza rufipogon*) showed heterozygous genotypes, although to a different extent (Figure 3A–C). Both the control plant Nipponbare and another example, Hexi 39, showed homozygous backgrounds (Figure 3D,E), although some small chromosome fragments display a heterozygous status. We also found another type of variety, Lvmi 3, which contains 1–5% of heterozygous chromosome fragments (Figure 3F). Additionally, some varieties such as Yinguang and Fengdao 11 show a large portion of heterozygous fragments, especially Fengdao 11 (Figure 3G,H). 

### 3.4. Analysis of the Genes Involved in Rice Pathogen Infections

The whole-genome SNP array was designed to cover most of the functional markers located in or near the cloned genes that are reported as important regulators involved in bio-stress resistance. For genes with more than one SNP marker existing in the gene, haplotypes were used to compare the genotypes of the tested varieties with representative alleles. For example, the haplotypes of the *Bph6*, *Bph9*, *Bph14*, *Bph15*, *Bph18* and *Bph26* genes are represented by 26, 26, 29, 56, 30 and 24 SNPs, respectively (Table 1). For genes with less nucleotide diversity, such as *Rymv1*, *Pi63*, *Pizt* and *STV11*, the functional SNP was used to distinguish the functional or non-functional alleles.

Generally, the favorable alleles of genes conferring resistance to different pathogens exhibit different status in the local varieties. The favorable alleles of genes related to brown planthopper resistance are almost absent in the tested varieties, except that only one variety (0.6%) shared the functional allele of *Bph18* with a representative variety (Table 1). For genes conferring rice blast resistance, the favorable alleles of *Pi1*, *Pid3* and *Pita* are the most used, but only accounted for 32.2%, 12.0% and 12.0% of all varieties, respectively (Table 1). The proportions of the favorable alleles of *Pi2*, *Pi5*, *Pia*, *Pid2*, *Pikh* and *Pi63* in all varieties are less than 10%. Additionally, the favorable alleles of *Pi9*, *Pigm* and *Pizt* are completely absent in all varieties. Five genes *xa13*, *Xa21*, *Xa23*, *xa5* and *Xa7* are commonly used for rice bacterial leaf blight resistance. However, only 20.3% of all varieties share favorable alleles as the representative variety (Table 1).

**Figure 4:**
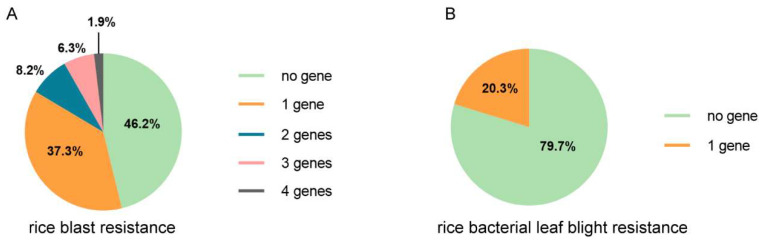
The summary of the existence of rice blast genes and rice bacterial leaf blight resistance genes in the local varieties in the Yunnan province. The proportion of varieties that harbor a different number of rice blast resistance genes (**A**) and rice bacterial leaf blight resistance (**B**).

To further understand the genetic background with regard to the bio-stress resistance in the collection of varieties, the number of favorable alleles possessed by different varieties was counted. About half of the varieties contain no favorable alleles of genes conferring rice blast resistance (Figure 4A), and more than half of the varieties contain no favorable alleles of genes conferring rice bacterial leaf blight resistance (Figure 4B). About 20% of the varieties harbor one favorable allele of genes with regard to rice bacterial leaf blight. No variety contains two or more favorable alleles. For genes conferring rice blast resistance, 37.3% of the varieties harbor one favorable allele, and 16.4% varieties harbor more than one, but at most four, favorable alleles (Figure 4A).

### 3.5. Analysis of the Genes Involved in Rice Eating and Cooking Qualities

The breeding of a variety with better eating and cooking qualities was another purpose of this breeding program. Thus, we analyzed the genotypes of the key genes *Wx* and *ALK* regulating amylose content as well as *BADH2*, which controls rice fragrance. Interestingly, 94.2% and 68.8% of our varieties possess the favorable alleles of *Wx* and *ALK*, respectively (Table 2). However, only one fifth of all varieties harbors the favorable allele of the fragrance gene *BADH2*. Further analysis revealed that the varieties containing the favorable alleles of both *Wx* and *ALK* account for only 35.1%. These results provide an overall view of the genetic constitution of the target traits, which may assist further breeding to increase favorable alleles together.

## 4. Discussion

The collection of varieties was clustered into three groups (Figure 2). Group II contained five varieties, including Yunhui 290, which is a classical *indica* inbred variety (https://ricedata.cn/variety/varis/606611.htm (accessed on 30 April 2022)). This result suggests that the other four varieties, sharing a more common background with Yunhui 290 in group II, may represent *indica* varieties. Likewise, the varieties of group III grouped together with Nipponbare may represent *japonica* varieties. Introduced varieties, such as Dozikara-1, Yuanbai and Linyou 22, are clustered in group III, but into different subgroups. This result implies that these introduced varieties have been well utilized and introgressed into local varieties, therefore creating several new varieties. However, the varieties Lvmi 3, Lvmi1, SMGR 4, Lvmi 2, Nanjing 5055 and Xuebai were clustered together, which suggests that these introduced varieties share a highly common background and have not been well utilized by local breeders.

The background analysis showed that the wild accessions *Oryza rufipogon*, *Oryza officinalis* and *Oryza meyeriana* contain different levels of heterozygous fragments in their genome (Figure 3), which suggests that these accessions still remain wild in nature, although they were collected and maintained by researchers for several years. Another finding in the background analysis showed that several tested varieties, such as Lvmi 3, possess only a few numbers of heterozygous fragments. This result not only intuitively exhibits the genetic background of the tested variety, but also shows the powerful function of SNP array in the breeding course. In fact, several studies showed that the whole-genome chip array has been well used for the deliberate selection of target genes [13,31].

Thanks to the advances in functional genomics in rice, a series of genes regulating bio-stress resistance were cloned, such as *Bph* genes for brown planthopper resistance [32], *Pi* genes for rice blast resistance [33] and *Xa* (*xa*) genes for rice bacterial leaf blight resistance [34]. Interestingly, our results show that the favorable alleles of bio-stress resistance genes are less retained in the local varieties in the Yunnan province. The favorable alleles of the *Bph* genes are almost absent in all the tested varieties, except one variety that contains the favorable allele of *Bph18* (Table 1). For genes with regard to rice bacterial leaf blight resistance, 20.3% of the varieties contain the favorable allele of *Xa21*, and no varieties harbor the favorable allele of *xa13*, *Xa23*, *xa5* and *Xa7*. These results reveal the fact that rice breeding during the last few decades pursued a high grain yield and paid less attention to bio-stress resistance. Nowadays, beyond obtaining a high grain yield, new varieties should be adapted to the growing conditions with less pesticides to meet the goals of sustainable agriculture. Thus, our results could provide essential information for both candidate parent and favorable allele selection for target genes in further breeding progress.

## 5. Conclusions

In order to obtain a better understanding of the genetic background of the collection of varieties cultivated in the Yunnan province, China, a collection of 154 varieties cultivated in that region were subjected to a genetic background analysis with high-density SNP array, which revealed the complicated genetic diversities represented by the distinguishing background and high percentage of homozygous genomic fragments. Further analysis showed that the favorable alleles of pathogen resistance genes displayed a relatively low frequency of existence in this collection of varieties. However, most varieties possessed the favorable alleles of *Wx* and *ALK* genes, which confer better eating and cooking qualities to rice. These results provide useful information for the future genetic improvement of rice in breeding practices.

## Figures and Tables

**Table 1:** Functional analysis of genes in response to bio-stress resistance in the collection of rice varieties.

Gene	Chr.	Type of Markers	Representative Variety	Marker(s)	Function	Percentages of Favorable Allele
*Bph14*	3	Haplotype	NA	29 SNPs	brown planthopper resistance	0%
*Bph15*	4	Haplotype	NA	56 SNPs	brown planthopper resistance	0%
*Bph18*	12	Haplotype	NA	30 SNPs	brown planthopper resistance	0.6%
*Bph26*	12	Haplotype	NA	24 SNPs	brown planthopper resistance	0%
*Bph6*	4	Haplotype	NA	26 SNPs	brown planthopper resistance	0%
*Bph9*	12	Haplotype	NA	26 SNPs	brown planthopper resistance	0%
*Pi1*	11	Haplotype	NA	10 SNPs	rice blast resistance	32.3%
*Pi2*	6	Haplotype	NA	99 SNPs	rice blast resistance	8.2%
*Pi5*	9	Haplotype	NA	33 SNPs	rice blast resistance	1.3%
*Pi9*	6	Haplotype	NA	80 SNPs	rice blast resistance	0%
*Pia*	11	Haplotype	NA	13 SNPs	rice blast resistance	0.6%
*Pid2*	6	Haplotype	NA	66 SNPs	rice blast resistance	3.8%
*Pid3*	6	Haplotype	NA	65 SNPs	rice blast resistance	12.0%
*Pigm*	6	Haplotype	NA	90 SNPs	rice blast resistance	0%
*pikh*	11	Haplotype	NA	34 SNPs	rice blast resistance	1.9%
*Pita*	12	Haplotype	NA	32 SNPs	rice blast resistance	12.0%
*Pi63*	4	SNP	Kahei	1 SNP	rice blast resistance	7.0%
*Pizt*	6	INDEL	Toride 1	1 Indel	rice blast resistance	0%
*xa13*	8	Haplotype	NA	61 SNPs	rice bacterial leaf blight resistance	0%
*Xa21*	11	Haplotype	NA	12 SNPs	rice bacterial leaf blight resistance	20.3%
*Xa23*	11	Haplotype	NA	40 SNPs	rice bacterial leaf blight resistance	0%
*xa5*	5	Haplotype	NA	48 SNPs	rice bacterial leaf blight resistance	0%
*Xa7*	6	Haplotype	NA	18 SNPs	rice bacterial leaf blight resistance	0%
*Rymv1*	4	SNP	Nipponbare	1 SNP	yellow mottle virus resistance	99.4%
*STV11*	11	SNP	Kasalath	1 SNP	rice stripe virus resistance	32.9%

**Table 2:** Functional analysis of the major genes affecting the eating and cooking qualities in the collection of rice varieties.

Gene	Chr.	Type of Markers	Representative Variety	Function	Percentages of Favorable Allele
*Waxy*	6	SNP	Nipponbare (favorable allele)	Increase in amylose content	94.2%
*ALK*	6	SNP	Minghui 63 (unfavorable allele)	Increase in amylose content	68.8%
*BADH2*	8	SNP	Suyunuo (favorable allele)	Grain fragrance	20.1%

## Data Availability

The datasets supporting the conclusions of this article are included within the article.
